# Empathy skill-dependent modulation of working memory by painful scene

**DOI:** 10.1038/s41598-017-04702-9

**Published:** 2017-07-03

**Authors:** Mo Chen, Yuan-Zheng Wang, Chen-Chen Ma, Qi-Ze Li, Han Zhou, Jie Fu, Qian-Qian Yang, Yong-Mei Zhang, Yu Liu, Jun-Li Cao

**Affiliations:** 1 Jiangsu Province Key Laboratory of Anesthesiology, Xuzhou Medical University, Xuzhou, Jiangsu 221004 China; 2 Jiangsu Province Key Laboratory of Anesthesia and Analgesia Application Technology, Xuzhou Medical University, Xuzhou, Jiangsu 221004 China; 3 Department of Anesthesiology, The Affiliated Hospital of Xuzhou Medical University, Xuzhou, Jiangsu 221002 China

## Abstract

As an important online information retaining and processing function, working memory plays critical roles in many other cognitive functions. Several long-term factors, such as age, addiction and diseases, have been affirmed to impair working memory, but whether or how the short-term factors, like painful stimuli or emotions, regulate the human working memory ability is not well explored. Here we investigated the influences of empathic pain on upcoming working memory and existing working memory, by presenting human subjects with the pictures depicting painful or neutral scene. After separating the subjects into two groups, the more empathic group and relatively indifferent group, according to a well-accepted questionnaire (the Interpersonal Reactivity Index (IRI)), the modulatory effect emerged. Empathic pain might exerted either a facilitating effect or an impairing effect, which was closely correlated with the personal empathy skills. Meanwhile, different aspects of subjects’ empathy traits exerted distinct effects, and female subjects were more vulnerable than male subjects. Present study reveals a new modulatory manner of the working memory, via empathy skill-dependent painful experience.

## Introduction

In 1956, after summarizing plenty pioneering researches, Miller concluded that human temporal information processing system had severe limitations^1^. In the following decades, this argumentation was verified by numerous well-designed experiments, which further quantified that the working memory capacity is around four chunks^2–4^.

As a core cognitive function, working memory highly relates with many brain functions, especially long-term memory^5–7^, intelligence^8–10^ and learning^11^. In addition, several long-term factors, including age^12^, addiction^13^ and diseases^14^, have been reported to cause changes in human working memory. But whether or how the short-term factors modulate working memory is not quite clear.

Among these short-term factors, physical pain and psychological pain, which serve as important indicators of the potential danger and effective guidance of our avoidance behaviors, could dramatically control cognitive functions or reallocate brain resources. But whether they influence working memory is still not well explored. There were several studies focusing on this issue, which showed that external interferences could impair working memory performances of human subjects, but these impairments should be largely attributed to the attentional interruption^15–18^.

Recently, a growing body of fMRI studies demonstrates that the empathic pain causes significant bilateral activity changes in several brain regions, including anterior midcingulate cortex and anterior insula, two key regions for own pain perception^19–23^. Due to this partial cerebral commonality between empathic pain and physical pain, we proposed that the empathic pain would also elicit changes in human working memory performance. We employed visual working memory task and presented the subjects with either painful pictures depicting other persons suffering from painful stimuli or neutral pictures, to study the effects of empathic pain on working memory.

Furthermore, in consideration of the different experience of each subject while viewing painful pictures, we introduced a well-accepted empathy questionnaire, the Interpersonal Reactivity Index (IRI)^24^. The IRI questionnaire provides a multi-dimensional assessment of empathy skills and has been demonstrated to exhibit good intrascale consistency, test–retest reliability and convergent validity by vast scientific studies^25–28^. The IRI questionnaire has also been translated into different languages, and has been proved to be adequate for examining empathy traits across people from various cultures^29–32^.

Present study found that the working memory performances were modulated when subjects witnessed pictures displaying others’ pain, and this effect correlated with personal empathy skills. The more empathic subjects (subjects with high IRI scores, high IRI group) and relatively indifferent subjects (subjects with low IRI scores, low IRI group) performed differently in present study. Basically, the more empathic subjects would bear an impairing working memory, and the relatively indifferent subjects would stay uninfluenced or bear a slightly facilitating effect. Furthermore, the phenomenon was prominent on female subjects, and was only moderate on male subjects.

## Results

### Painful pictures elicited empathic pain

**Figure 1:**
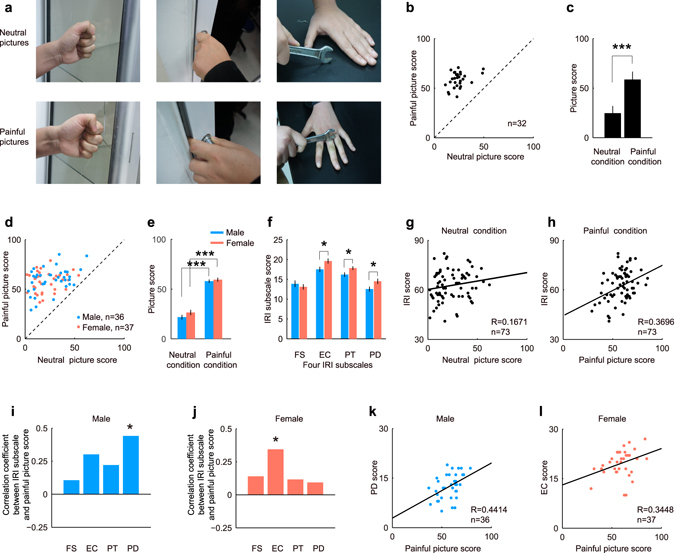
Examples of the pictures used to elicit empathic pain and the subjects’ responses. **(a)** Three example pairs of pictures. In total, 32 pairs of photographs were used, and each pair contained one painful picture and one corresponding neutral picture. **(b)** Subjects’ subjective evaluation of the empathic pain scores of every picture pair. Each dot represents the mean empathic pain scores of the painful and neutral pictures in one pair. **(c)** The empathic pain scores of painful pictures were significantly higher than those of the neutral pictures (*P* < 0.001, d.f. = 31, paired *t*-test, *d* = 4.3133). **(d)** The empathic pain scores of the painful and neutral pictures evaluated by the male (blue dots) and female (red dots) subjects. **(e)** Both male and female subjects gave higher marks to the painful pictures than to the corresponding neutral pictures (male, *P* < 0.001, d.f. = 35, *d* = 3.0333; female, *p* < 0.001, d.f. = 36, *d* = 2.2921, paired *t*-test). **(f)** An empathy questionnaire, the Interpersonal Reactivity Index (IRI) was employed to measure the empathy skills of individual subjects. IRI has four seven-item subscales, including fantasy (FS), empathic concern (EC), perspective-taking (PT) and personal distress (PD). Females displayed higher scores than males on the last three subscales, EC, PT and PD, but not on FS (FS, *P* = 0.47, d.f. = 71, *d* = 0.17; EC, *P* = 0.0213, d.f. = 71, *d* = 0.5511; PT, *P* = 0.0284, d.f. = 71, *d* = 0.5239; PD, *P* = 0.0354, d.f. = 71, *d* = 0.502, *t*-test). **(g,h)** Subjects exhibiting higher IRI scores generally gave higher marks to both neutral **(g)** and painful **(h)** pictures, but the IRI scores only significantly correlated with the painful picture scores (R = 0.3696, *P* = 0.0013). The solid lines represent linear regression. **(i–l)**, The IRI subscale, PD, significantly correlated with the painful picture scores for males (**i**,**k**, R = 0.4414, *P* = 0.007); While another subscale, EC, significantly correlated with the painful picture scores for females (**j**,**l**, R = 0.3448, *P* = 0.0366).

To study the effects of empathic pain on working memory, 32 pairs of photographs were used. Each pair consisted of one painful picture and one corresponding neutral picture (Fig. 1a)^19^. A picture evaluation task was designed, in which subjects gave their subjective evaluations of the empathic pain degrees of all 64 pictures. Generally, subjects gave higher scores to painful pictures than to neutral pictures, across all 32 pairs (Fig. 1b). The scores of painful pictures were significantly higher than those of corresponding neutral pictures (Fig. 1c; *P* < 0.001, d.f. = 31, paired *t*-test, *d* = 4.3133. Mean ± s.d.: neutral, 24.3319 ± 7.4992; painful, 58.4017 ± 8.2791). In total, 73 subjects participated in the picture evaluation task, and all of them evaluated painful pictures as having higher empathic pain degrees, compared with neutral pictures (Fig. 1d; Male, n = 36; Female, n = 37). Empathic pain degrees of the pictures (F(1, 142) = 249.1676, *P* < 0.001, two-way ANOVA), but not genders exerted significant influence on picture scoring (Fig. 1e). Both males and females gave statistically higher scores to painful pictures than to neutral pictures (Male, *P* < 0.001, d.f. = 35, *d* = 3.0333; Female, *P* < 0.001, d.f. = 36, *d* = 2.2921, paired *t*-test), but there was no score difference between genders, under either neutral or painful condition.

In present study, three indexes were calculated: the correct rate difference, the mean correct rate, and the mean correct rate difference. The correct rate difference was calculated as the correct rate under neutral condition minus the correct rate under painful condition, and was used to measure whether working memory performance was affected by painful photographs. The mean correct rate was the mean value of correct rates of set size 6 and 10, and was employed to compared working memory performances while the set sizes were large (set size 6 and 10). The third index, the mean correct rate difference, was computed first by calculating the mean correct rate of set size 6 and 10 under neutral condition and painful condition separately, and then calculated the difference between two conditions (neutral minus painful). This is a summarizing index and was designed to compare the influences at large set sizes (set size 6 and 10).

### Subjects exhibiting higher IRI scores generally gave higher scores to painful pictures

To quantitatively assess the individual empathy skills, an empathy questionnaire, the Interpersonal Reactivity Index (IRI) was administered to subjects, which provides a self-report survey of individual empathy traits^24^. The IRI questionnaire consists of 28 items, which fall into four subscales: fantasy (FS), which measures the subjects’ ability to imaginatively transpose themselves into the fictitious characters in books, movies or plays; Second, empathic concern (EC), which assesses the tendency to experience the feelings of warmth, compassion and concern for unfortunate people; Third, perspective taking (PT), which measures subjects’ willingness to adopt perspectives of others; Finally, personal distress (PD), which denotes the tendency to experience personal anxiety and unease when witnessing unfortunate events.

Generally, female subjects exhibited higher scores than male subjects on total score of the IRI questionnaire (F(1, 284) = 7.3102, *P* = 0.0073, two-way ANOVA), which was consistent with previous study^24^. Specifically, females scored significantly higher than males on the last three IRI subscales, EC, PT and PD, but not on FS (Fig. 1f; FS, *P* = 0.47, d.f. = 71, *d* = 0.17; EC, *P* = 0.0213, d.f. = 71, *d* = 0.5511; PT, *P* = 0.0284, d.f. = 71, *d* = 0.5239; PD, *P* = 0.0354, d.f. = 71, *d* = 0.502, *t*-test). Furthermore, subjects’ self-report IRI scores correlated with their performances in the picture evaluation task: subjects exhibiting higher IRI scores generally gave higher scores to both neutral and painful pictures. But IRI scores only significantly correlated with painful picture scores (Fig. 1h, R = 0.3696, *P* = 0.0013), rather than with neutral picture scores (Fig. 1g, R = 0.1673, *P* = 0.1577). The correlations between four IRI subscales and painful picture scores showed coherent trends: the correlation coefficients were positive among all four IRI subscales, for both males (Fig. 1i) and females (Fig. 1j). Additionally, the PD scores significantly correlated with the painful picture scores for males (Fig. 1k, R = 0.4414, *P* = 0.007), and the EC scores significantly correlated with the painful picture scores for females (Fig. 1l, R = 0.3448, *P* = 0.0366).

### The influences of empathic pain on upcoming working memory

**Figure 2:**
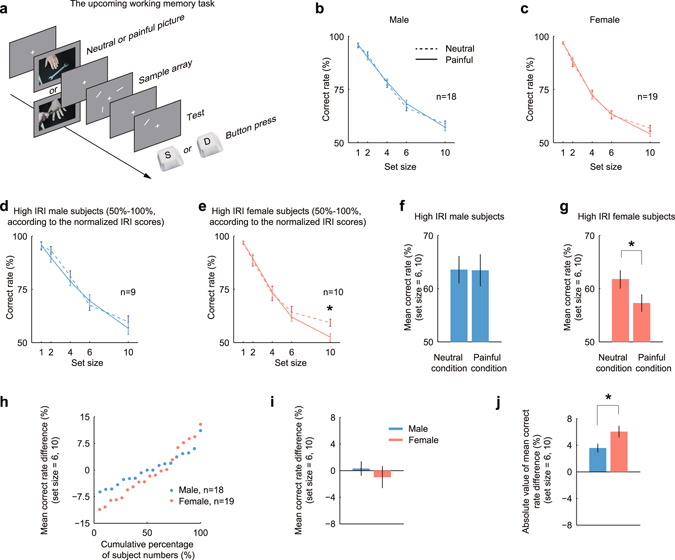
The influences of empathic pain on the upcoming working memory. **(a)** The upcoming working memory task tested the influences of painful pictures on the working memory of events which would occur in the future. After the fixation point disappeared, either a neutral picture or a painful picture would appear on the screen center randomly. The sample array appeared later which might consist of 1, 2, 4, 6 or 10 bars. After a working memory period, a test bar appeared and the subjects should indicate whether the orientations of the sample and test bars were the same or different by pressing “S” or “D” button accordingly. **(b,c)** The performances of males **(b)** and females **(c)** in upcoming working memory task. The correct rate decreased as the number of items (set size) in the sample array increased. There was no statistical difference between neutral (dashed line) and painful conditions (solid line) for either gender. (**d–g)** The performances of high IRI male subjects **(d,f)** and high IRI female subjects **(e,g)**. Subjects were arranged by their IRI scores from small to large, and high IRI subjects referred to the first 50% subjects. While the number of items was large (set size = 6, 10), the correct rates of females **(e)**, but not those of males **(d)**, were impaired by empathic pain. For males, the mean correct rate of large set sizes (set size = 6, 10) did not differ between the neutral and painful conditions (**f**). For females, the mean correct rate of large set sizes (set size = 6, 10) under painful condition decreased 7.3%, compared with neutral condition (**g**, F(1, 44) = 4.4788, *P* = 0.04, two-way ANOVA). **(h–j)** The mean correct rate differences between neutral and painful conditions (neutral minus painful) at large set sizes distributed differently between genders. The females exhibited relatively more dispersed distribution **(h)**. The averaged mean correct rate difference did not differ much between males and females (**i**), but the absolute value of mean correct rate difference of females was significantly higher than that of males (**j**, *P* = 0.0314, d.f. = 35, *t*-test, *d* = 0.7375).

In present study, we tested two aspects of the influences of empathic pain on working memory: the influences on upcoming working memory events (the upcoming working memory task) and the influences on existing working memory events (the existing working memory task). In the upcoming working memory task, painful or neutral pictures were presented before the sample array onset. The time sequence was illustrated in Fig. 2a: each trial began with a central fixation point appearing on the screen. After the fixation point offset, either a neutral picture or a painful picture appeared randomly, and subjects were instructed to watch the picture. Subsequently, the subjects’ working memory performances were tested by presenting them with a sample array, which randomly consisted of 1, 2, 4, 6 or 10 bars. Subjects should memorize both orientation and location of each bar as accurately as possible. After a working memory period, a test bar appeared in one of the locations where the sample bars had appeared. The orientation of test bar was randomly chosen, which would be either the same as sample bar in the same location or be different with a certain orientation difference. The subject indicated same or different by pressing “S” button or “D” button accordingly. In the upcoming working memory task, three orientation differences were tested, 30° (13 subjects, Male, n = 6, Female, n = 7), 40° (12 subjects, Male, n = 6, Female, n = 6) and 60°(12 subjects, Male, n = 6, Female, n = 6).

The correct rates of both males (Fig. 2b) and females (Fig. 2c) decreased as the number of objects in sample array (set size) increased, in consistence with previous findings^2, 5^. When we compared performances between neutral condition (Fig. 2b,c, dashed lines) and painful condition (Fig. 2b,c, solid lines), neither males or females showed any significant difference at any set size.

Interestingly, after the IRI questionnaire was introduced and subjects were divided into more empathic group (high IRI group, subjects who got top 50% in the IRI questionnaire) and relatively indifferent group (low IRI group, subjects between 0% and 50%) (see Materials and Methods), the performance profiles altered. Although the performances of high IRI males did not differ much between neutral and painful conditions (Fig. 2d), the correct rates of high IRI females under painful condition decreased at large set sizes (set size 6 and 10), compared with neutral condition (Fig. 2e). Especially at set size 10, the correct rate under painful condition was significantly lower than that under neutral condition (mean ± s.e.m.: neutral, 59.3148% ± 1.7116%; painful, 52.5811% ± 1.5628%; *P* = 0.0085, d.f. = 11, paired *t*-test, *d* = 1.1356). In this task, since three groups of subjects were recruited and participated in the task with different orientation differences (30°, 40° or 60°), the IRI scores were normalized within each group to eliminate sampling bias (see Materials and Methods).

We computed the mean correct rate of set size 6 and 10 for high IRI male subjects and high IRI female subjects separately: the mean correct rates did not statistically differ between two conditions for high IRI male subjects (Fig. 2f, F(1, 32) = 0.002, *P* = 0.9647, two-way ANOVA), but the mean correct rate under painful condition was significantly lower than that under neutral condition for high IRI female subjects (Fig. 2g, F(1, 44) = 4.4788, *P* = 0.04, two-way ANOVA). These results indicate that, in upcoming working memory task, the working memory ability of males is generally not influenced by empathic pain, but females exhibiting high IRI scores are susceptible to empathic pain, and their working memory performances are impaired while the working memory load (set size) is large.

Meanwhile, the performances of females fluctuated more strongly than those of males: the mean correct rate difference (neutral minus painful) at large set sizes (set size 6 and 10) distributed more dispersed for females than for males (Fig. 2h). The mean correct rate differences of both genders distributed around 0 (Fig. 2i), but the absolute value of mean correct rate difference for females was significantly higher than that for males (Fig. 2j, *P* = 0.0314, d.f. = 35, *t*-test, *d* = 0.7375). These findings reveal that the female subjects, but not the male subjects, are vulnerable to empathic pain in the upcoming working memory task.

### The correlations between personal empathy skills and upcoming working memory performances

**Figure 3:**
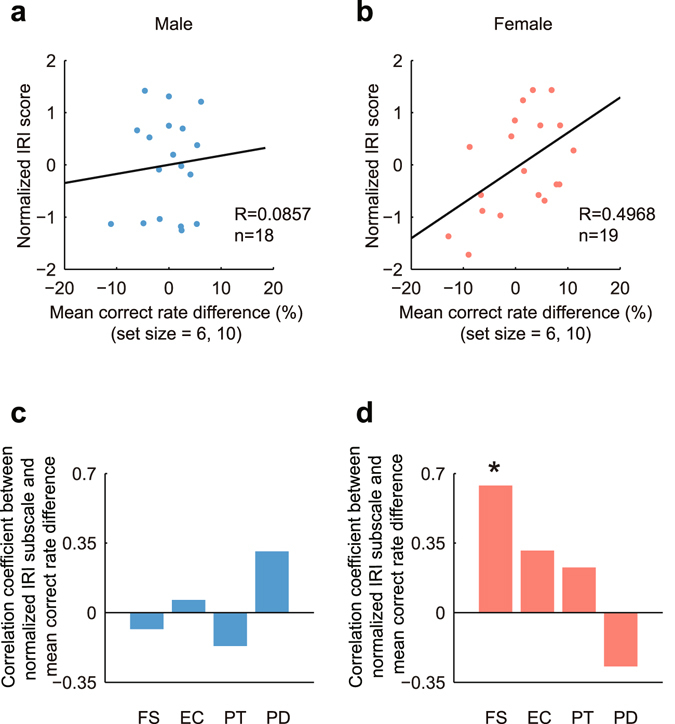
Empathy skill-dependent modulation of working memory in the upcoming working memory task. **(a,b)** The correlations between normalized IRI scores and mean correct rate differences for males **(a)** and females **(b)**. The mean correct rate differences significantly correlated with normalized IRI scores for females (R = 0.4968, *P* = 0.0305), but not for males (R = 0.0857, *P* = 0.7353). Solid lines represent linear regression. **(c,d)** The correlations between four IRI subscales and the mean correct rate differences. For males, only PD showed a relatively high, but non-significant correlation **(c)**. For females, FS significantly correlated with the mean correct rate differences (**d**, R = 0.6384, *P* = 0.0033).

The influences of empathic pain on working memory were closely related to subjects’ empathy traits. Figure 3a and b depicted the mean correct rate differences of large set sizes against normalized IRI scores: while males only exhibited a weak positive correlation (Fig. 3a, R = 0.0857, *P* = 0.7353), females exhibited a significant positive correlation (Fig. 3b, R = 0.4968, *P* = 0.0305). These results suggest that the higher the female subjects scored in IRI questionnaire, the more severe the impairment of empathic pain on working memory might be. When we looked into the details and analyzed four IRI subscales, the influences were complex. For males, there was no significant correlation among all subscales, and only PD showed a relatively high positive correlation (Fig. 3c). For females, the correlations of first three subscales were positive, and FS exhibited a significant positive correlation (Fig. 3d; FS, R = 0.6384, *P* = 0.0033). The FS subscale (fantasy) reflects subjects’ ability to imaginatively transpose themselves into fictitious characters. For female subjects, the significant correlation of FS indicates that females who could easily get involved in fictional situations might be impaired more severely by empathic pain in the upcoming working memory task.

### The influences of empathic pain on existing working memory

**Figure 4:**
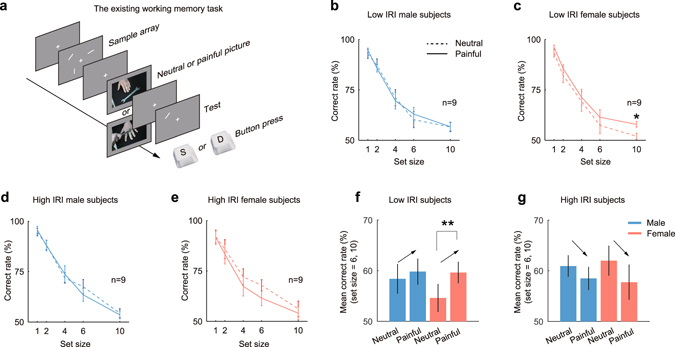
The influences of empathic pain on the existing working memory. **(a)** The existing working memory task tested influences of painful pictures on the working memory of events which occurred in the past, so painful or neutral pictures were presented after the sample array onset. The sample array still randomly consisted of 1, 2, 4, 6 or 10 bars. The working memory period was the time interval between sample array offset and test bar onset. **(b,c)** The performances of low IRI male subjects **(b)** and low IRI female subjects **(c)**. For the low IRI male subjects, there was no statistical difference between neutral (dashed line) and painful conditions (solid line). For the low IRI female subjects, the correct rates under the painful condition were higher than those under the neutral condition across all set sizes, and the difference was significant at set size 10 (*P* = 0.0153, d.f. = 16, *t*-test, *d* = 1.2792). **(d,e)** The performances of high IRI male subjects **(d)** and high IRI female subjects **(e)**. Generally, the working memory at large set sizes (set size = 6, 10) was impaired for both genders. **(f)** In summary, for the low IRI subjects, the mean correct rates (set size = 6, 10) under painful condition were higher than those under neutral condition for both genders, and the difference was significant for females (*P* = 0.0079, d.f. = 8, paired *t*-test, *d* = 0.6802). **(g)** For the high IRI subjects, the mean correct rates (set size = 6, 10) under painful condition were lower than those under neutral condition for both genders, but neither of these differences was significant. Arrows are only for illustration purpose.

Being different from the upcoming working memory task, the existing working memory task was designed to study influences of empathic pain on working memory of existing events. In this task (Fig. 4a), either a neutral picture or a painful picture would appear after the sample array disappeared, and subjects were still freely to watch the picture. A test bar appeared later to test subjects’ working memory performances. In the existing working memory task, three orientation differences were tested, 30° (Male, n = 6; Female, n = 6), 60° (Male, n = 6; Female, n = 6) and 90° (Male, n = 6; Female, n = 6).

Findings in the existing working memory task complemented those in the upcoming working memory task. The major finding was that empathic pain affected working memory performances of low IRI subjects and high IRI subjects in an opposite way. Note that the subjects were still divided into low IRI group and high IRI group by the normalized IRI scores (Conventions are as those in the upcoming working memory task).

For the low IRI male subjects, the performances under neutral or painful conditions did not show statistical difference (Fig. 4b). However, for the low IRI female subjects, the correct rates under painful condition were higher than those under neutral condition among all set sizes (Fig. 4c), and the correct rates at set size 10 were significantly different (mean ± s.e.m.: neutral, 51.9603% ± 1.5275%; painful, 57.8277% ± 1.3501%; *P* = 0.0153, d.f. = 16, paired *t*-test, *d* = 1.2792). When we considered the high IRI subjects, the phenomena were reversed. For high IRI male subjects, their performances under neutral or painful conditions still did not show significant difference (Fig. 4d). But for high IRI female subjects, the performances under painful condition were lower than those under neutral condition, at all set sizes except 1 (Fig. 4e).

The phenomena were more obvious when we compared mean correct rates of large set sizes (set size 6 and 10). Within the low IRI group, the mean correct rates under painful condition were higher than those under neutral condition for both genders (Fig. 4f), and females exhibited a significant difference (*P* = 0.0079, d.f. = 8, paired *t*-test, *d* = 0.6802). Within high IRI group, the mean correct rates under painful condition were lower than those under neutral condition for both genders (Fig. 4g). Results suggest that empathic pain exerts a facilitating effect on low IRI subjects and a slight (non-significant) impairing effect on high IRI subjects.

**Figure 5:**
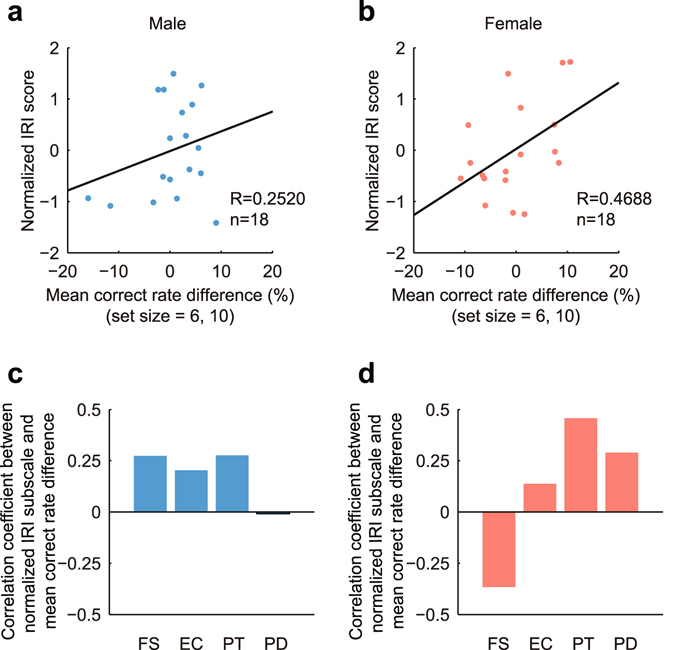
Empathy skill-dependent modulation of working memory in the existing working memory task. **(a,b)** The correlations between normalized IRI scores and mean correct rate differences for males **(a)** and females **(b)**. The correlation was significant for females (R = 0.4688, *P* = 0.0497), but not for males (R = 0.2520, *P* = 0.3131). **(c,d)** The correlations between four IRI subscales and the mean correct rate differences. For males, the first three subscales, FS, EC and PT, showed relatively high, but non-significant correlations, and the fourth subscale, PD, almost exhibited no correlation. **(c)** For females, no significant correlation was found (**d**).

In the existing working memory task, the mean correct rate differences at large set sizes also correlated with normalized IRI scores. For both genders the correlations were positive, and the correlation of females was significant (Fig. 5a, male, R = 0.2520, *P* = 0.3131; Fig. 5b, female, R = 0.4688, *P* = 0.0497). The correlations between four IRI subscales and the mean correct rate differences were shown in Fig. 5c and d. For males, there were non-significant positive correlations on first three IRI subscales, FS, EC and PT, and was a slightly negative correlation on the PD subscale (Fig. 5c). For female subjects, the correlation of FS subscale was negative, and this was contrary to the positive correlation of FS in upcoming working memory task (Fig. 5d). Overall, the correlation analyses in existing working memory task implicate that the working memory performances of high IRI subjects are more likely to be impaired by empathic pain, while the working memory performances of low IRI subjects generally bear a facilitating effect.

## Discussion

In present study, we addressed the modulatory effects of empathic pain on working memory, by presenting subjects with photographs depicting painful scenes, either before or after the working memory events. Basically, we found that: (1) the modulatory effects were stronger on female subjects, but were only moderate on male subjects; (2) empathic pain exerted the influences via an empathy skill-dependent way; and (3) different aspects of the empathy traits played distinct roles.

Various psychological studies have found that females score significantly higher than males in the measures of empathic tendencies, which suggests that females generally perform more empathically than males^24, 33^. In present study, females also exhibited higher scores in IRI questionnaire than males. While exposuring to the painful photographs, the working memory performances of males and females were modulated to different extents.

In one fMRI study conducted by Singer and colleagues, they measured the brain activity while subjects observed the confederates receiving painful stimulation^27^. The empathy-related brain activation was significantly reduced in males while they passively observed a cue indicating that a defector was receiving pain. The authors concluded that the empathic responses in males are modulated by valuation of other people’s social behavior, which could “indicate a predominant role for males in the maintenance of justice”. In present study, female subjects generally experienced stronger modulatory effects on their working memory, no matter facilitated (low IRI subjects) or impaired (high IRI subjects), in both tasks. This should be largely attributed to females’ good empathy skills - they transferred themselves deeper into the painful scene, and were more vulnerable. Male subjects, in contrast, with less empathic responses, would stay relatively uninfluenced viewing painful scenes. Together with the fMRI study^27^, males and females, with inherently different empathy skills, react differently to empathy-related events, and might eventually lead to different prosocial roles.

The IRI questionnaire assesses both the cognitive dimension (PT and FS) and the affective dimension (EC and PD) of empathy^24^, and different studies emphasize and utilize different IRI subscales accordingly. As independent measures of cognitive dimension and emotional dimension respectively, PT and EC are the most frequently employed subscales^34–36^. In present study, four IRI subscales played distinct functional roles in two working memory tasks.

In the picture evaluation task, PD showed significant correlation with the painful piture scores for males, and EC showed significant correlation with the painful piture scores for females. Those results are likely to reflect different natures of the evaluation process by two genders: the males rely on their personal distress to evaluate an painful scene, that is, how much distress or discomfort they experience in response to others’ misfortune may primarily determine how painful they estimate an painful scene; in contrast, the females mainly rely on their empathic concern ability, that is, the more compassion and concern they feel for others, the more painful they evaluate an painful scene to be.

In the upcoming and existing working memory tasks, we did not observe any significant or consistent correlation between IRI subscales and working memory performances of males. Nevertheless, the performances of females revealed interesting phenomena: in the upcoming working memory task, the correlation coefficient of FS was positive and the correlation coefficient of PD was negative, but the trends were reversed in existing working memory task, that is, the correlation coefficient of FS was negative and the correlation coefficient of PD was positive. As explained by Decety and Jackson, an essential role of empathy is to understand or feel other person’s emotional state, without confusion of self- and other-feelings^37^. According to this point of view, people with high fantasy ability (FS) and low personal distress (PD), to some extent, would possibly have better social interactions. In present study, working memory of those subjects (high FS and low PD) was impaired in upcoming working memory task, and was facilitated in existing working memory task. All conclusions are based on observations from present study, and further experiments are needed to confirm these phenomena.

Studies have shown that acute pain impairs performances in working memory tasks^16–18^, and this effect is more prominent on females^15^. As a distressing experience associated with actual or potential injury, pain interrupts attention^38^, and therefore might impair working memory. Contrary to this point of view, the data from present study suggest that the influences of empathic pain on working memory might be impairing or facilitating, depending not only on the paradigm design, but also on the individual empathy skills.

Briefly, present study provides a behavioral window exploring the modulatory mechanism of empathic pain on working memory. In the EEG study conducted by Vogel and Machizawa^39^, they observed stepwise amplitude increase of event-related potentials (ERPs) as the number of items in memory array increases, and the amplitude of this activity approaches a plateau determined by each individual’s memory capacity. Similarly, we expect to see that the stepwise amplitude increase or the activity plateau would be modulated by empathic pain, and this need to be further tested.

## Methods

### Subjects

In total, 73 healthy subjects were recruited (age 20–35; 36 males, 37 females). Two subjects were the authors and others were naïve to the purpose of this study. All subjects signed the Informed Consent Form before participating in experiments. Experimental procedures were performed in accordance with the WHO’s Standards and the operational guidance for ethics review of health-related research with human participants and were approved by the key laboratory of anesthesiology, Xuzhou Medical University, Jiangsu Province.

### Apparatus

Visual stimuli were presented on a 21-inch monitor controlled by a computer running MATLAB (MathWorks) with Psychtoolbox (PTB-3; Brainard and Pelli). The vertical refresh rate of the monitor was 120 Hz, and the spatial resolution was set at 1024 × 768 pixels. Seated in a light-isolated room, the subject viewed monitor from a distance of 80 cm using a chin and forehead rest to stabilize head position. Luminance was measured with a photometer (ST-86LA; Photoelectric instrument factory of Beijing Normal University). The eye position was monitored at a sample rate of 1 kHz using a video-oculography eye tracker (EyeLink 1000 Desktop; SR Research), which was controlled by MATLAB software. All data analyses were done with MATLAB version R2009b.

### Visual stimuli and eye position detection

Screen background was uniformly illuminated at 1.6 cd/m^2^. The fixation point was a white orthogonal cross (0.5° in length) and was illuminated at 14.2 cd/m^2^. The sample and test bars were white rectangles (0.3 × 0.7°), and were illuminated at 14.2 cd/m^2^. Gaze position was recorded from 200 ms before the fixation point disappeared to the end of a trial. A trial would be aborted if the subject broke fixation, that is, the recorded eye position fell out of an invisible 5 × 5° square window centered at the fixation point during fixation period.

### Measure of the empathy skills

The empathy traits of the subjects were measured using the interpersonal reactivity index (IRI questionnaire)^24^, translated to Chinese. The IRI questionnaire consists of 28 items, which fall into four subscales: fantasy (FS), the tendency to transpose oneself into fictitious characters; empathic concern (EC), “other-oriented” feelings of sympathy and compassion for others in need; perspective taking (PT), the tendency to adopt others’ opinions; personal distress (PD), “self-oriented” negative feelings in response to others’ distress.

### General procedure

The major aim of present study was to investigate influences of empathic pain on working memory. The empathic pain was elicited by presenting subjects with painful or neutral pictures. We shot 32 pairs of photographs, with each pair consisting of one painful picture and one corresponding neutral picture (64 pictures in total). Generally, in each pair of pictures, the painful picture showed photograph of hands which were suffering from or under the potential hurt from harmful stimuli, such as needle, fire, broken glass and so on. The corresponding neutral picture was taken in the same environment, and we kept everything in these two pictures as similar as possible, except that the harmful stimulus was far from the hand or was absent from current scene in the neutral picture condition.

The whole study consisted of 4-day experiments, 3 sessions per day. The total testing time for each subject was <60 min per day. Experiments were divided into sessions, each of which lasted for 10–15 min, followed by a resting interval of 5–10 min. On the first day, subjects participated in picture evaluation task. On days 2–4, subjects performed either upcoming working memory task or existing working memory task. At the end of day 4, subjects completed the IRI questionnaire.

### The picture evaluation task

In this task, subjects reported their subjective evaluation of the empathic pain degree of each picture. Trials began with the appearance of a white cross, the fixation point, on the screen center. Subjects were required to fixate the fixation point for at least 500 ms, and 500 ms after the fixation point disappeared a picture was presented on the screen center. The picture was either a painful or a neutral photograph, and the presentation duration was randomized among 50, 200, 500, 1000, 1500, 2000 and 3000 ms. A ruler then appeared on the screen, 500 ms after the picture disappeared. Subjects were informed to indicate the empathic pain degree of previous picture on the ruler by moving a vertical bar, using the left and right arrow keys. The empathic pain degree on the ruler ranged from “pain free” to “severe pain”, and was converted to 0–100 scores according to the ruler position.

### The upcoming working memory task

The upcoming working memory task was designed to study the effects of empathic pain on working memory of upcoming events, so the painful or neutral picture appeared before sample array onset. In this task, a fixation point first appeared on the screen center. Around 400–600 ms after the fixation point disappeared, either a painful or a neutral picture would appear randomly on the screen center for 1900–2100 ms, during which subjects could freely move their eyes. A sample array then appeared (duration, 200 ms; 700 ms after the picture disappeared), which randomly consisted of 1, 2, 4, 6 or 10 bars. There were 12 possible bar locations, which were evenly separated and located at equal eccentricity (5°) around fixation point. Then the sample array disappeared, and after a memory period of 1800–2200 ms, a test bar appeared at one of the locations where previous sample bars had appeared. The orientation of test bar was randomly chosen, which would be either the same as sample bar in the same location, or be different with a certain orientation difference. In present study, three orientation differences were tested (30°, n = 13; 40°, n = 12; 60°, n = 12). Subjects compared the orientation of test bar with the orientation of sample bar in the same location, and responded by pressing “S” or “D” button for the same or different condition, respectively.

### The existing working memory task

The existing memory task tested the effects of empathic pain on existing working memory. A painful or neutral picture appeared for 400–500 ms, 400–600 ms after the sample array offset. The memory period was set the same as that in upcoming working memory task, that is, 1800–2200 ms after sample array offset, the test bar appeared. Also, three orientation differences were tested (30°, n = 12; 60°, n = 12; 90°, n = 12). Other parameters in this task were the same as those in upcoming working memory task.

### The normalization of IRI scores

In both upcoming and existing working memory tasks, three different conditions were tested (30°, 60° and 90°). Three groups of subjects were recruited in each task and one subject only participated in one condition. To avoid sampling bias, that is, the subjects in one condition exhibiting especially high or low IRI scores, the IRI scores of each group were normalized within group by computing the *z*-score. All subjects were then arranged by their normalized IRI scores from small to large, and were divided into high IRI group (50–100%) and low IRI group (0–50%), accordingly. Unless otherwise specified, the high IRI group or high IRI subjects referred to the first 50% subjects according to the normalized IRI scores, and the low IRI group or low IRI subjects referred to the last 50% according to the normalized IRI scores, respectively.

### The correlation analysis

Correlation coefficients were computed using Pearson’s correlation coefficient method. The mathematic formula is:

1 $${\rm{CC}}=\frac{{\sum }_{{\rm{i}}}({{\rm{x}}}_{{\rm{i}}}-\overline{{\rm{x}}})({{\rm{y}}}_{{\rm{i}}}-\overline{{\rm{y}}})}{\sqrt{{\sum }_{{\rm{i}}}{({{\rm{x}}}_{{\rm{i}}}-\overline{{\rm{x}}})}^{2}{\sum }_{{\rm{i}}}{({{\rm{y}}}_{{\rm{i}}}-\overline{{\rm{y}}})}^{2}}}$$

Both correlation coefficient and statistic *p* value were computed by matlab build-in function *corrcoef*.
